# 

**Published:** 1910-11-05

**Authors:** 


					P LLBttAjiV. ,
rSWRGt# r,?.KERAP5?<t!vi
K V.
)i 1?. "^10
Telecraoh* Add THE MODERN MEDICAL
telegraphic Address: AND Telephone Number
Ospedale, London. INSTITUTIONAL JOURNAL. "" G.rrard.
HEW SERIES. No. 195, Vol. VIII.
No. 1267, Vol. XLIX. ?ATURDAY,
Nov. 5th, 1910.
PRICE THREEPENCE

				

